# Dental status of an institutionalized elderly population of 60 years and over in Qingdao, China

**DOI:** 10.1007/s00784-015-1581-6

**Published:** 2015-09-11

**Authors:** Qian Zhang, Qian Jing, Anneloes E. Gerritsen, Dick J. Witter, Ewald M. Bronkhorst, Nico H. J. Creugers

**Affiliations:** Department of Oral Function and Prosthetic Dentistry, College of Dental Science, Radboud University Nijmegen Medical Centre, Philips van Leydenlaan 25, 6525 EX Nijmegen, The Netherlands; Department of Prosthodontics, Yantai Stomatological Hospital, North street 142#, Yantai City, Shandong Province People’s Republic of China; Department of Preventive and Restorative Dentistry, College of Dental Science, Radboud University Nijmegen Medical Centre, Philips van Leydenlaan 25, 6525 EX Nijmegen, The Netherlands

**Keywords:** Dental status, DMF, Tooth replacements, China, Institutionalized older people

## Abstract

**Objectives:**

The aim of this study was to investigate dental status of institutionalized elders and to relate outcomes with background variables and oral functionality.

**Materials and methods:**

Dental status of 512 elders (≥60 years) from eight nursing homes in Qingdao were analyzed in terms of prevalence of decayed (D), missing (M), filled (F), and replaced teeth (R). Multivariate logistic regression was applied to determine relationships with the background variables age, gender, and SES. Prevalence of D, M, and F was analyzed also for separate dental regions. For determining oral functionality, prevalence of dentitions with ≥20 teeth without and with tooth replacements was plotted against age.

**Results:**

Mean number of D varied from 3.8 at 60 years to 4.6 at 90 years, M from 3.6 at 60 years to 6.7 at 90 years for the lower jaw, and from 3.0 at 60 years to 8.0 at 90 years for the upper. Mean number of F in each jaw was low: 0.2 at 60 years to 0.4 at 90 years. Gender and SES effects were limited. Molars had significantly higher prevalence of D and M than premolar and anterior teeth. Seventy percent of participants of 60 years had ≥20 natural teeth and 12 % at 90 years. Including tooth replacements, 96 % at 60 years, and 84 % at 90 years had ≥20 teeth.

**Conclusions:**

In this sample of institutionalized elders, dental status of the majority of participants did not represent a functional dentition without tooth replacements.

**Clinical relevance:**

Institutionalized Chinese elders showed relatively low numbers of decayed teeth but high numbers of missing teeth.

## Introduction

China’s low mortality rate, combined with the one-child policy, has resulted in a dramatic ageing of China’s overall population. Today, the population of older people in China is the largest in the world with approximately 165 million people (approximately 13 %) being 60 years and over, and 9 % being 65 years and over. It is estimated that the population of 60 years and over will increase up to 400 million people by 2050 [1]. Of these people, the majority is still embedded in the traditional Chinese family culture, which is based on great respect for elders and an informal care system. Nevertheless, due to demographic changes, there is a nationwide shift from traditional care for elders to institutionalized care [2]. Currently, approximately 1.5 % of older people in China live in nursing homes and apartments for older people, but it is expected that these figures will increase in the coming years [3].

Although oral disease is recognized as one of the major problems in public health and is a good indicator for general health, data on the prevalence of dental diseases amongst elders in China is very scarce, and for institutionalized elders, restricted to some data from Hong Kong [4–8]. The most recent 3rd Chinese National Oral Epidemiological Survey, published in 2008, reported a relatively low prevalence of decayed teeth and a high prevalence of missing teeth, whilst filled teeth were almost absent for people aged 65–74 [9]. Data on dental status of Chinese people above 80 years is even lacking [10]. Knowledge of the prevalence of dental diseases affecting older elders provides insight into future dental needs of the elderly population.

The number of missing teeth is related with the functionality of dentition and might therefore indicate possible (future) dietary insufficiencies [6, 11, 12]. To meet an acceptable level of oral function, the World Health Organization (WHO) has formulated a strategic goal to preserve dentitions comprising at least 20 natural teeth for life [13]. For Japanese elderly populations, it has been shown that having at least 20 teeth at the age of 80 is a discriminating indicator for better oral condition as well as for better health status [14, 15]. For assessing oral functionality, however, a systematic review emphasized the relevance of teeth present in different dental regions, i.e. the anterior, the premolar, and the molar region [16]. The authors concluded that for adequate oral function and satisfaction with the dentition, molars are considered dispensable as long as 9–10 occluding pairs of teeth retain, including a complete anterior region and some premolars. As a consequence, it is considered relevant to analyze dental status also for the different dental regions separately.

Dental status as expressed by decayed, missing, and filled teeth is considered to reflect past and present dental disease, as well as past and present treatment modalities and utilization of oral health care. DMFT as the most often used measure of dental status describes disease and disease effects in a single score. In contrast, the separate scores of the components D (number of decayed teeth), M (number of missing teeth), and F (number of filled teeth) relate to disease, disease effects, and subsequent treatment. For this reason, as a proxy of past and present oral health care, the present study is focusing on the DMF components separately. Replacement of missing teeth also refers to subsequent treatment of disease aiming at raising the level of oral functionality. Therefore, in the present analysis of dental status, replaced teeth were also considered.

With analyzing dental status and replaced teeth of different dental regions and applying the ‘at least 20 teeth’ criterion, it is aimed to relate effects of received dental treatment with oral functionality. Therefore, the aim of this study was to investigate dental status in Chinese institutionalized older people with respect to decayed, missing, filled, and replaced teeth and to relate the outcomes to social-demographic variables and oral functionality.

## Materials and methods

### Sample construction

The present study was conducted in 2012 in Qingdao City, located at the east coast of Shandong Province, Eastern China. A purposive sample of eight elderly homes (varying from 33 to 359 residents; total number of residents = 1226) in different geographic districts in Qingdao City was selected on the basis of accessibility and convenience. Information on the purpose and procedures of the study was provided to the management of the homes and their residents. It was aimed to include 500 participants in this study.

Residents were visited room by room and invited to participate. In total, 512 people (which is 42 % of the total population of the visited elderly homes) capable of communication and with no life threatening condition agreed to participate. The number of participants per nursing home ranged from 7 (21 %) to 171 (86 %); 66 % of the participants were females. The study was carried out in compliance with the Helsinki Declaration. Prior to start of the study, the Ethics Committee of the Affiliated Hospital of Medical College, Qingdao University, approved the study protocol.

### Questionnaire and clinical oral examination

According to the study protocol, oral informed consent was obtained from all participants before they entered the study. First, a structured questionnaire was completed. This questionnaire has been used in previous studies, and included questions regarding social-demographic variables (age, gender, and socio-economic status (SES)) [6]. The questionnaires were completed by the participants, but if (functional) illiteracy, visual impairment, or expressive language disorders made that difficult, the questions were read aloud and answers were recorded by the investigator, or, if present, a family member or nursing home staff member assisted in completing the questionnaire. After completion of the questionnaire, it was checked for unrecorded items and if applicable, participants were requested to complete the form.

For assessment of SES (high, middle, low), a modified Kuppuswamy classification was used [17], which is based on the subject’s level of education (5 levels: higher education; college; primary school; no formal education, literate; no formal education, illiterate), previous occupation (3 levels: (1) white collar: office worker, teacher, doctor, academic researcher, government officers; (2) service people: salespeople, house worker, vehicle driver; (3) blue collar: farmer, factory worker, forestry worker, fisher, (lower) military personal), and income (4 levels: (1) income covers expenses, no loans needed; (2) income does not cover expenses, no loans needed; (3) income covers expenses, loans needed incidentally; (4) income does not cover expenses, loans needed regularly).

Next, participants underwent an oral examination. Two dentists trained by an experienced researcher performed the examinations. Inter-observer agreements amongst the experienced researcher and the two observers for assessing decayed teeth, missing teeth, filled teeth, and replaced teeth were good (all kappas ≥0.8). Examinations were done by using a mouth mirror and a probe.

Procedures and diagnostic criteria recommended by the World Health Organization were applied [18]. Teeth were neither cleaned nor dried before clinical examination, but food debris obscuring visual inspection was removed.

Caries was assessed by visual inspection; if any doubt existed, caries was recorded as not present. ‘Filled’ teeth with secondary caries were recorded as ‘decayed’. Teeth without being decayed or filled were considered ‘sound’. Retained roots were considered in the analyses in two different ways. According to the WHO criteria, a retained root was considered as a decayed tooth. In the analysis of tooth replacements, however, retained roots were considered candidates for replacement and therefore considered as missing teeth. Tooth replacements were recorded as such if teeth were replaced either by fixed or removable dental prostheses.

### Data analyses

The analyses included the following dependent variables: decayed (D), missing (M), filled (F), and replaced teeth (R). First, for dentate participants, mean DMFT was calculated and mean numbers of the D, M, and F components and sound teeth were plotted against age.

Next, to determine relationships of the background variables age, gender, and SES, with D, M, F, and R, multivariate logistic regression analyses were applied. For this analysis, people edentulous in one or both jaws were excluded and the variables D, M, F, and R were dichotomized: 0 = no decayed, no missing, no filled, no replaced teeth, respectively; 1 = one or more decayed, one or more missing, one or more filled, or one or more replaced teeth, respectively. In this analysis, only participants having decayed, missing, or filled teeth (D + M + F > 0) were included.

Thirdly, mean DMFT scores and mean scores of D, M, and F components were determined for each dental region. For each region, relative scores for the D, M, and F components were determined by dividing the number of teeth having the respective status (e.g. decayed) by the total number of D + M + F teeth in that region. For within-subject comparisons, mean relative scores for D, M, and F for the molar regions were compared with the other dental regions using paired *t*-tests.

Finally, to visualize the relation between age and oral functionality by assessing the proportion of participants with ≥20 teeth, a line through the scatter plot between age and that proportion was fitted using third degree polynomials. This was calculated separately for teeth present and for teeth present plus tooth replacements. Edentulous people were excluded for this analysis. Also, participants older than 90 years (*n* = 20) were excluded in the scatter plots because of low numbers of participants per year of age.

## Results

Of the 512 participants, 360 (70 %) reported their perceived general health as being fair to excellent, whereas 152 (30 %) reported their health as being poor.

More than half (62 %) of the participants were 80 years and older (Table 1). Out of the 512 participants, 128 (25 %) were edentulous in one or both jaws, of which 58 (11 % of the total sample) were edentulous in both jaws. In the youngest age group (60–69 years), 6 % were edentulous in one or both jaws; in the oldest age group (≥80 years), this was 34 %. In total, 384 participants were dentate in each jaw, which is 75 % of the sample.

**Table 1:** Number (%) of participants, % female participants, and number (%) of participants dentate in each jaw according to age group

Age groups	Participants	% Female	Dentate in each jaw
60–69	47 (9)	66	44 (94)
70–79	150 (29)	70	131(87)
≥80	315 (62)	64	209 (66)
Total	512 (100)	66	384 (75)

### Decayed, missing, and filled teeth

The majority of the participants in each jaw had one or more decayed (68.2 % in the youngest age group to 90.4 % in the oldest) and/or one or more missing (95.5–98.1 %) teeth. The percentage of participants with one or more filled teeth ranged from 11.4 to 23.0; the percentage of participants with one or more replaced teeth varied from 34.1 to 56.5 (Table 2). The overall mean DMFT score was 15.12 ± 8.17.

**Table 2:** Percentage of participants dentate in each jaw (*n* = 384) with decayed, missing, and filled teeth, and with teeth replaced

Number of participants per age category		Percentage of participants with teeth:			
		Decayed	Missing	Filled	Replaced
60–69	44	68.2	95.5	11.4	34.1
70–79	131	82.4	97.7	23.7	61.8
≥80	209	90.4	98.1	23.0	56.5

**Fig. 1:**
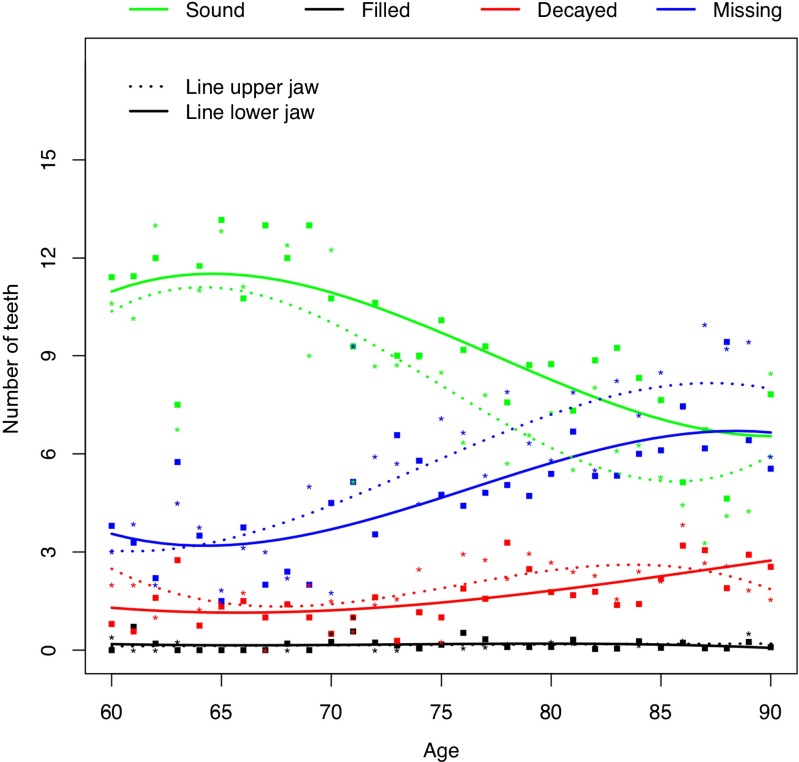
Mean number of decayed, missing, filled, and sound teeth in upper and lower jaw by age (*n* = 434 dentate participants 60–90 years of age)

By and large, the mean number of D was similar for the upper and lower jaw (Fig. 1). In the upper jaw, the mean number of D varied slightly from 2.0 at the age of 60 to 1.5 at the age of 90; in the lower jaw, the mean number of D varied from 0.8 for subjects aged 60 years to 2.5 for those at 90 years. For both jaws combined, the mean number of D varied slightly from 3.8 at 60 years to 4.6 at 90 years of age.

The mean number of M in the lower jaw varied from 3.6 at the age of 60 to 6.7 at the age of 90; for the upper jaw, these numbers were 3.0 and 8.0, respectively. Participants aged 64 and over had higher mean numbers of M in the upper jaw than in the lower.

The mean number of F in each jaw was low, ranging from 0.2 for 60 year-old participants in the lower jaw to 0.4 for 90 year-old participants, also in the lower jaw. The mean number of sound teeth varied from 11.1 in the lower jaw of 60-year-old participants to 4.1 in the upper jaw for 90-year-old participants. The mean number of sound teeth was higher in the lower jaw than in the upper.

### Background variables and dental regions

Multivariate logistic regression analysis (Table 3) showed a higher chance for decayed teeth (D) at higher age for the entire dentition as well as for the dental regions separately (all ORs ≥1.037 per additional year of age, all *p* values ≤ 0.028). Age was positively associated with M for the anterior region (OR = 1.044 per year, *p* = 0.015) but not with M for the premolar and molar regions (*p* values ≥0.070). Age was not significantly associated with F (*p* values ≥0.416) but positively associated with R for the entire dentition (OR = 1.028 per year; *p* = 0.044) and for the molar region (OR = 1.036 per year; *p* = 0.012). Females had a higher chance for D for the entire dentition (OR = 2.17; *p* = 0.012) and for the molar region (OR = 1.60; *p* = 0.037). These gender associations were also found for F: for the entire dentition, OR = 3.00 (*p* < 0.001), and for the molar region, OR = 3.03 (*p* = 0.002). Participants in the category SES_high_ had significantly higher chance for F (OR = 2.34; *p* = 0.001). This was the case for the premolar and molar regions but not for the anterior region. The variables D, M, and R were not associated with SES.

**Table 3:** Odds ratios for D, M, F, and R teeth, 95 % confidence intervals (CI), and level of significance for the entire dentition, and in the anterior, premolar, and molar regions separately (*n* = 384)

	D			M			F			R		
	Adjusted OR	*p* value	95 % CI	Adjusted OR	*p* value	95 % CI	Adjusted OR	*p* value	95 % CI	Adjusted OR	*p* value	95 % CI
Entire dentition (*n* = 383)^a^												
Age^b^	1.085	*<0.001*	[1.046…1.126]	1.048	0.287	[0.962…1.141]	1.013	0.467	[0.979…1.048]	1.028	*0.044*	[1.001…1.056]
Female^c^	2.17	*0.012*	[1.19…3.95]	0.62	0.558	[0.12…3.12]	3.00	*<0.001*	[1.64…5.47]	1.01	0.947	[0.66…1.57]
SES-high^d^	1.44	0.271	[0.75…2.75]	0.82	0.785	[0.19…3.51]	2.34	*0.001*	[1.4…3.92]	1.08	0.734	[0.70…1.66]
Anterior region (309)^a^												
Age^b^	1.038	*0.028*	[1.004…1.073]	1.044	*0.015*	[1.008…1.081]	1.013	0.682	[0.952…1.078]	1.008	0.636	[0.975…1.043]
Female^c^	1.10	0.724	[0.65…1.85]	0.75	0.327	[0.43…1.33]	2.74	0.077	[0.90…8.39]	1.13	0.646	[0.67…1.89]
SES-high^d^	0.69	0.155	[0.41…1.15]	1.05	0.859	[0.60…1.84]	1.47	0.401	[0.60…3.59]	1.23	0.440	[0.73…2.07]
Premolar region (*n* = 296)^a^												
Age^b^	1.039	*0.026*	[1.005…1.075]	1.04	0.070	[0.997…1.084]	0.980	0.503	[0.923…1.04]	1.011	0.511	[0.978…1.046]
Female^c^	1.39	0.207	[0.83…2.31]	1.24	0.513	[0.65…2.39]	2.47	0.085	[0.88…6.93]	0.84	0.509	[0.50…1.41]
SES-high^d^	0.98	0.944	[0.59…1.64]	0.95	0.891	[0.49…1.85]	2.51	*0.032*	[1.08…5.83]	1.03	0.924	[0.62…1.71]
Molar region (*n* = 380)^a^												
Age^b^	1.037	*0.010*	[1.009…1.066]	1.062	0.171	[0.975…1.156]	1.017	0.416	[0.977…1.059]	1.036	*0.012*	[1.008…1.065]
Female^c^	1.60	*0.037*	[1.03…2.50]	0.27	0.224	[0.03…2.23]	3.03	*0.002*	[1.48…6.20]	0.96	0.841	[0.62…1.48]
SES-high^d^	1.12	0.634	[0.71…1.75]	1.44	0.662	[0.28…7.29]	2.55	*0.002*	[1.42…4.59]	0.86	0.512	[0.56…1.34]

Italicized figures indicate statistical significance

^a^Difference in numbers due to exclusion of participants with D + M + F = 0 (intact dentition or region)

^b^Per year

^c^Reference (OR=1) is male

^d^Reference (OR=1) is SES middle/low

Mean DMFT score for the molar region was 7.52 ± 2.83; for the premolar region, this was 3.28 ± 2.65, and for the anterior region, 4.31 ± 3.70. Within-subject comparisons showed that the relative scores for the D component for the molar regions in upper and lower jaw were significantly lower, but that the relative scores for the M component were significantly higher than for the premolar and anterior regions (Table 4). Differences in relative scores for the F components were relatively small amongst the dental regions and significant only for the comparison of the upper molar region with the upper anterior region (lower score for upper molar region (*p* = 0.007)).

**Table 4:** Relative scores (%) for decayed, missing, and filled teeth in molar (Mol), premolar (PMol), and anterior (Ant) dental regions, and mean difference (%) of relative scores between the dental regions (*n* = 384)

	Number^a^	Comparison	Molar score	Comparative score	Difference	95 % CI	*p* value
Upper jaw							
Decayed							
	256	Mol-PMol	16.5	34.4	−17.9	[−22.5…−13.4]	*<0.001*
	264	Mol-Ant	16.0	51.6	−35.6	[−40.9…−30.3]	*<0.001*
Missing							
	256	Mol-PMol	82.3	62.9	19.3	[14.8…23.9]	*<0.001*
	264	Mol-Ant	83.3	44.7	38.5	[33.3…43.7]	*<0.001*
Filled							
	256	Mol-PMol	1.3	2.7	−1.4	[−3.2…0.4]	0.132
	264	Mol-Ant	0.7	3.7	−2.9	[−5.0…−0.8]	*0.007*
Lower jaw							
Decayed							
	229	Mol-PMol	21.0	40.3	−19.2	[−24.6…−13.9]	*<0.001*
	224	Mol-Ant	19.9	37.2	−17.3	[−23.3…−11.4]	*<0.001*
Missing							
	229	Mol-PMol	75.8	55.9	19.9	[14.3…25.5]	*<0.001*
	224	Mol-Ant	77.3	61.0	16.3	[10.1…22.4]	*<0.001*
Filled							
	229	Mol-PMol	3.1	3.8	−0.7	[−3.0…1.6]	0.562
	224	Mol-Ant	2.9	1.8	1.1	[−1.0…3.2]	0.318

Italicized figures indicate statistical significance

^a^Difference in numbers due to exclusion of participants with D + M + F = 0 (intact entire dentition or dental region)

**Fig. 2:**
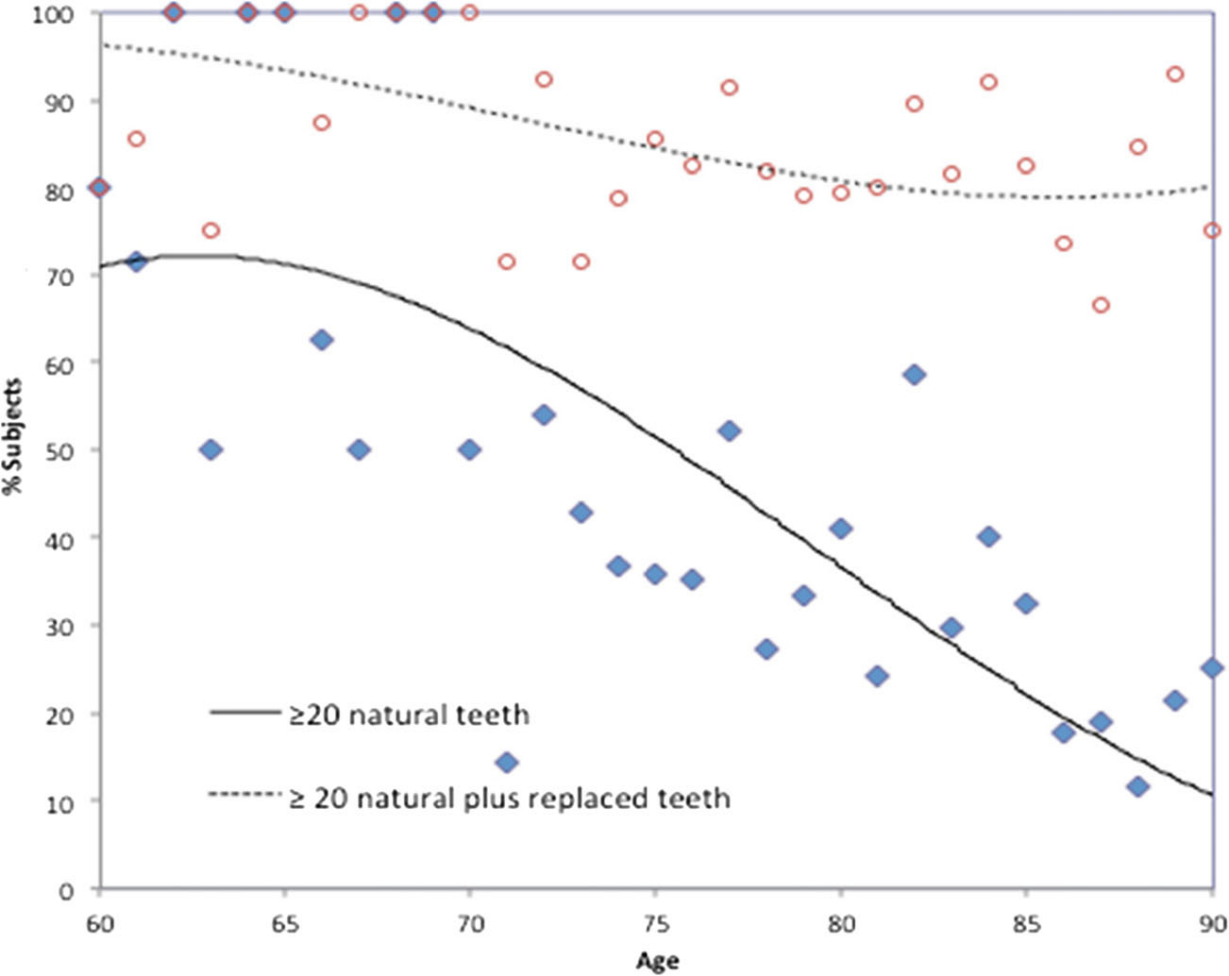
Percentage of dentate participants (*n* = 434 dentate participants 60–90 years of age) with at least 20 natural teeth or with at least 20 natural plus replaced teeth

The percentage of dentate participants that presented at least 20 natural teeth varied from 70 at the age of 60 to 12 at the age of 90 (Fig. 2). When replaced teeth are taken into account (at least 20 natural plus replaced teeth), these percentages were 96 at 60 years and 84 at the age of 90.

## Discussion

### Sample

As this sample of 512 institutionalized elders was a convenient sample, concerns of representation and bias may rise. The eight nursing homes involved can be considered representative for Qingdao, but this city cannot be considered representative for China. Qingdao has had faster economic development and urbanization during the last 20 years than China as a whole. Although not representative for China, we consider the outcomes of the present study as valuable information for developing oral health care strategies for institutionalized older people. Bias might evolve from differences in nursing home sizes and different participation rates for each nursing home. However, to our knowledge, there were no systematic differences between the small and large nursing homes, such as population compositions and care facilities. Also, we could not observe any relationship between participation rates per nursing home and its size.

### Decayed, missing, and filled teeth

The study shows an age gradient with higher numbers of missing teeth amongst elder participants (Fig. 1). This finding is in line with data from a cross-sectional study amongst community dwelling older people in Qingdao city [19] (both studies report approximately seven missing teeth at the age of 70 years). However, the number of missing teeth in the present study was lower than in another study amongst a community dwelling population in Qingdao area (approximately 10 at the age of 70), which also included people living in rural areas [6].

Looking at the different dental regions, more often, the molar region relatively showed missing teeth than the premolar and anterior regions; decayed teeth in this region were relatively less prevalent. With respect to filled teeth, the differences between the regions were not significant, except for the upper jaw where the anterior region relatively more often had filled teeth than the molar region. These findings together might indicate a different treatment modality for molars and point at the direction of extraction of decayed molars rather than restoration. Such a difference in treatment modality between anterior teeth and molars might result not only from more severe decay in molars, but also from a higher prevalence of periodontal disease in the molar region. Prevalence of periodontitis in elderly Chinese people has been reported in 22 % of 65–74-year-old people and only in 14 % of people 75 and over [20]. This relatively low periodontitis prevalence in older elders might point at extractions for periodontal reasons [21].

### Utilization of dental care

In this sample, the relationships between decayed and filled teeth and between missing and replaced teeth reflect the present and past utilization of dental care. The relatively high prevalence of tooth decay is neither balanced by the percentage of participants with filled teeth nor with the number of filled teeth (Table 2, Fig. 1). This indicates a low grade of restorative caries lesion treatment. On the other hand, a relatively high grade of tooth replacement can be noted: the low percentage of participants with at least 20 natural teeth is fairly ‘compensated’ by the high percentage of people that met the 20 teeth criterion when replaced teeth are taken into account. In a Japanese study amongst dentate older people, 48 % of subjects aged 60–69 years had less than 20 teeth; for subjects aged 80 years and over, this percentage was 78 [22]. However, that report did not provide replacement data.

In contrast with a previous report [23], it cannot be substantiated that females had a higher grade of utilization of dental care: females in the present study presented with similar prevalence of missing teeth and tooth replacements as males. Moreover, the higher prevalence of filled teeth in females was accompanied with a higher prevalence of decayed teeth. The role of SES in utilization of dental care in the present study is unclear: participants with SES_high_ showed a higher prevalence of filled teeth; however, SES was not associated with decayed and missing teeth, or with tooth replacements. The latter confirms findings of another study on the relationship between SES and utilization: amongst institutionalized older people in Japan, a social gradient in dental prosthesis utilization could be observed; however, the poorest income group showed prosthesis use as high as the highest income group [24]. In the present sample, given the high number of missing teeth, we consider the utilization of dental care aiming at tooth preservation during life as low. It seems that the dental care as provided in the past has been confined to replacement of teeth rather than to restorative treatment of decayed teeth. The findings of the present study implicate that the utilization of the dental care did not serve the WHO strategic goal of the preservation of a functional dentition comprising at least 20 natural teeth for life.

As mentioned in the introduction, there are almost no data on older elders and elders living in nursing homes from Mainland China. With the increasing number of elders living in nursing homes and given the limitation of the present study, a nationwide survey in China amongst these people is recommended.

## Conclusion

In this sample of institutionalized elders, the majority presented with a reduced dentition. Amongst elder participants, high numbers of missing teeth were found. The missing component of DMFT in the molar region was significantly larger than in the premolar and anterior regions. Dental status indicates that previous dental treatment has been confined mainly to tooth extraction and tooth replacement. This practice did not serve the WHO strategy of preserving functional dentitions comprising at least 20 natural teeth for life.
